# Antibiotic Resistance of *Pseudomonas aeruginosa* in Pneumonia at a Single University Hospital Center in Germany over a 10-Year Period

**DOI:** 10.1371/journal.pone.0139836

**Published:** 2015-10-02

**Authors:** Josef Yayan, Beniam Ghebremedhin, Kurt Rasche

**Affiliations:** 1 Witten/Herdecke University, Witten, Department of Internal Medicine, Division of Pulmonary, Allergy and Sleep Medicine, HELIOS Clinic, Wuppertal, Germany; 2 Witten/Herdecke University, Witten, Institute of Medical Laboratory Diagnostics, Center for Clinical and Translational Research, HELIOS Clinic, Wuppertal, Germany; University of Birmingham, UNITED KINGDOM

## Abstract

**Background:**

*Pseudomonas aeruginosa* is a common cause of community-acquired and nosocomial-acquired pneumonia. The development of resistance of *P*. *aeruginosa* to antibiotics is increasing globally due to the overuse of antibiotics. This article examines, retrospectively, the antibiotic resistance in patients with community-acquired versus nosocomial-acquired pneumonia caused by *P*. *aeruginosa* or multidrug-resistant (MDR) *P*. *aeruginosa*.

**Methods:**

Data from patients with community-acquired and nosocomial-acquired pneumonia caused by *P*. *aeruginosa* and MDR *P*. *aeruginosa* were collected from the hospital charts at the HELIOS Clinic, Witten/Herdecke University, Wuppertal, Germany, between January 2004 and August 2014. An antibiogram was created from all study patients with community-acquired and nosocomial-acquired pneumonia caused by *P*. *aeruginosa* or MDR *P*. *aeruginosa*.

**Results:**

A total of 168 patients with mean age 68.1 ± 12.8 (113 [67.3% males and 55 [32.7%] females) were identified; 91 (54.2%) had community-acquired and 77 (45.8%) had nosocomial-acquired pneumonia caused by *P*. *aeruginosa*. Patients with community-acquired versus nosocomial-acquired pneumonia had a mean age of 66.4 ± 13.8 vs. 70.1 ± 11.4 years [59 vs. 54 (64.8% vs. 70.1%) males and 32 vs. 23 (35.2% vs. 29.9%) females]. They included 41 (24.4%) patients with pneumonia due to MDR *P*. *aeruginosa*: 27 (65.9%) community-acquired and 14 (34.1%) nosocomial-acquired cases. *P*. *aeruginosa* and MDR *P*. *aeruginosa* showed a very high resistance to fosfomycin (community-acquired vs. nosocomial-acquired) (81.0% vs. 84.2%; 0 vs. 85.7%). A similar resistance pattern was seen with ciprofloxacin (35.2% vs. 24.0%; 70.4% vs. 61.5%), levofloxacin (34.6% vs. 24.5%; 66.7% vs. 64.3%), ceftazidime (15.9% vs. 30.9; 33.3% vs. 61.5%), piperacillin (24.2% vs. 29.9%; 44.4% vs. 57.1%), imipenem (28.6% vs. 27.3%; 55.6% vs. 50.0%), piperacillin and tazobactam (23.1% vs. 28.6%; 44.4% vs. 50.0%), tobramycin (28.0% vs. 17.2%; 52.0% vs. 27.3%), gentamicin (26.4% vs. 18.2%; 44.4% vs. 21.4%), and meropenem (20.2% vs. 20.3%; 42.3% vs. 50.0%). An elevated resistance of *P*. *aeruginosa* and MDR *P*. *aeruginosa* was found for cefepime (11.1% vs. 23.3%; 25.9% vs. 50.0%), and amikacin (10.2% vs. 9.1%; 27.3% vs. 9.1%). Neither pathogen was resistant to colistin (*P* = 0.574).

**Conclusion:**

While *P*. *aeruginosa* and MDR *P*. *aeruginosa* were resistant to a variety of commonly used antibiotics, they were not resistant to colistin in the few isolates recovered from patients with pneumonia.

## Introduction

*Pseudomonas* is a rod-shaped, aerobic, Gram-negative bacterium belonging to the family *Pseudomonadaceae* [1]. *Pseudomonas aeruginosa* easily adapts to the environment it inhabits and can also colonize and invade a human host to cause serious infections [2,3]. *P*. *aeruginosa* isolates that cause infections are thought to express various virulence factors. This pathogen is one of the most common causes of pneumonia [4,5].

Risk factors for the development of infections caused by *Pseudomonas* include neutropenia, cystic fibrosis, severe burns, and foreign device installations [2,3]. The general human population is refractory against infections caused by *Pseudomonas* species, but *Pseudomonas* species are physiologically highly flexible and able to act as opportunistic pathogens in humans with weakened immune systems [6]. *P*. *aeruginosa* causes life-threatening community-acquired pneumonia, nosocomial infections such as pneumonia, urinary tract infections, and bacteremia; and chronic lung infections in patients with cystic fibrosis [7]. The identification of the microbiological cause of a case of pneumonia is especially important for preservation of the sensitivity of bacteria to antibiotics and for regulation of drug therapy [7].

Pneumonia due to *Pseudomonas* can be transmitted in hospitals by nursing staff, medical equipment, sinks, disinfectants, and food. *Pseudomonas* infections are a serious problem in hospitals for two reasons. First, patients who are critically ill can die from pneumonia caused by *Pseudomonas*. Second, the elimination of *P*. *aeruginosa* in patients with infections is very difficult because of its resistance to a variety of antibiotics [7]. *P*. *aeruginosa* currently shows resistance to the following antibiotics: penicillin G; aminopenicillin, including those combined with beta-lactamase inhibitors; first and second generation cephalosporins; piperacillin; piperacillin and tazobactam; cefepime; ceftazidime; aminoglycosides; the quinolones; and the carbapenems; as well as colistin and fosfomycin [8]. Therefore, distinguishing the trends in resistance of *P*. *aeruginosa* becomes important for choosing the right antibiotic because *P*. *aeruginosa* is the fourth most common cause of pneumonia [9].

The increasing resistance of *P*. *aeruginosa* to numerous antibiotics, as a result of excessive antibiotic administration, is now leading to the accumulation of antibiotic resistance and cross-resistance between antibiotics and the appearance of multidrug-resistant (MDR) forms of *P*. *aeruginosa* [10]. The treatment of MDR *P*. *aeruginosa* pneumonia in critical patients is therefore becoming more of a challenge. These findings stress the importance of microbiologists providing clinicians with accurate information regarding the sensitivity patterns of antibiotics, so that clinicians can select an appropriate antibiotic for the timely treatment of infectious diseases while still helping to prevent the occurrence of resistance of *P*. *aeruginosa* to antibiotics.

For these reasons, the present investigation was conducted to identify antibiotics to which *P*. *aeruginosa* has developed resistance in the last 10 years. Data from the hospital database at the HELIOS Clinic, Witten/Herdecke University, in Wuppertal, Germany, were collected for all patients with pneumonia caused by *P*. *aeruginosa* and MDR *P*. *aeruginosa* who presented with clinical symptoms, such as cough with purulent sputum, shortness of breath, and fever; with elevated infection parameters in blood lab tests; and with evidence of infiltrates in chest X-rays, according to the International Classification of Diseases (ICD J15.1). The aim of this study was to determine the changes in antibiotic resistance according to susceptibility testing of tracheal or bronchial secretions and blood cultures of patients with community-acquired and nosocomial-acquired pneumonia caused by *P*. *aeruginosa* or MDR *P*. *aeruginosa* over a period of 10 years. Antibiotic use and failure of antibiotic treatment were monitored in the entire study population during the study period. Tackling pneumonia sooner with the correct choice of effective antibiotic against *P*. *aeruginosa* or MDR *P*. *aeruginosa* right from the start of treatment should shorten the duration of patients’ suffering and the length of their hospital stays, as well as reduce patient mortality.

## Material and Methods

### Ethics Statement

This study was conducted in accordance with the approved institutional guidelines of the Witten-Herdecke University in Germany. All patient data were anonymized prior to analysis. The Ethics Committee of the Witten-Herdecke University in Germany approved this study and all experimental protocols. Due to the retrospective nature of the study protocol, the Ethics Committee of the Witten-Herdecke University in Germany waived the need for written, informed consent.

### Patients

This quality-control observational study retrospectively examined the resistance to antibiotics in patients with diagnosed community-acquired or nosocomial-acquired pneumonia triggered by *P*. *aeruginosa* or MDR *P*. *aeruginosa*. The data were collected from hospital charts at the HELIOS Clinic, Witten/Herdecke University, in Wuppertal, Germany, in the study period from January 1, 2004 to August 12, 2014. The outcomes of susceptibility testing were compared between patients with community-acquired and nosocomial-acquired pneumonia due to either *P*. *aeruginosa* or MDR *P*. *aeruginosa*. Thus, all patients with community-acquired pneumonia caused by *P*. *aeruginosa* or MDR *P*. *aeruginosa* constituted the study group, while the patients with nosocomial-acquired pneumonia caused by *P*. *aeruginosa* or MDR *P*. *aeruginosa* were the reference group. The study population with community-acquired and nosocomial-acquired pneumonia initiated by *P*. *aeruginosa* or MDR *P*. *aeruginosa* was mixed in terms of age. All patients over 18 years of age who had community-acquired or nosocomial-acquired pneumonia caused by *P*. *aeruginosa* or MDR *P*. *aeruginosa* were included in the study.

### Setting

This study included all patients with pneumonia caused by *P*. *aeruginosa* or MDR *P*. *aeruginosa* who were treated on the general wards of all departments, intensive care units, infectious diseases settings, and at the Division of Pulmonary, Allergy and Sleep Medicine at the HELIOS Clinic in Wuppertal. The HELIOS Clinic Wuppertal, located in the two districts of Barmen and Elberfeld in northern Germany, is a maximum care teaching hospital of Witten/Herdecke University in Witten, with 24 departments and 967 beds. It offers almost the entire range of medical services for about 50,000 inpatients and 100,000 outpatients per year. The majority of the patients with pneumonia were treated at the Division of Pneumology at HELIOS Clinic. The Division of Pneumology, Allergology, Sleep, and Respiratory medicine offers a total of 70 beds with intensive care as a special department for respiratory medicine in various forms of respiratory failure, sleep disorders, and a special focus on acute and chronic benign as well as malignant diseases of the respiratory system. All patients with nosocomial-acquired pneumonia caused by *P*. *aeruginosa* or MDR *P*. *aeruginosa*, but who were treated initially for other medical reasons in other departments, such as Internal Medicine and Surgery, were included in this study. Those with *P*. *aeruginosa* or MDR *P*. *aeruginosa* infection that led to other infections, such as urinary infection, urosepsis, gastroenteritis, and meningitis, were excluded from the study. All patients examined at the Department of Neurology who had been suspected of having pneumonia caused by *P*. *aeruginosa* or MDR *P*. *aeruginosa* were excluded from this study because of restricted access to their patient data.

### Data collection

All patients admitted to this hospital during this study period who had a microbiological examination of tracheal or bronchial aspirates, blood cultures, and secretion of drainage performed for suspected pneumonia were included in this retrospective study. We routinely recorded the patient’s age and sex, the number of tracheal or bronchial aspirates, blood cultures, or secretion of drainage collected, the result of the culture, the susceptibility and resistance of the isolates to commonly used antimicrobial agents, comorbidities from the hospital records, inflammatory markers from the blood laboratory values. Data from these records were subsequently entered into an Excel (Microsoft) spreadsheet.

### Definition of Pneumonia

Pneumonia is an acute inflammation of the lung, primarily affecting the alveoli, which is usually caused by *P*. *aeruginosa* or MDR *P*. *aeruginosa*. Typical clinical symptoms of pneumonia include cough, chest pain, fever, and difficulty breathing. The diagnosis of pneumonia is performed by X-ray examination and sputum culture [11,12].

Community-acquired pneumonia caused by *Pseudomonas* is an acute infection of the lung parenchyma acquired from normal social contact within the community; this is in contrast to nosocomial-acquired pneumonia caused by *Pseudomonas*, which is acquired during hospitalization [11,12]. The classification of pneumonia caused by *Pseudomonas* was made in each case, from 2004 to 2014, according to the latest edition of the ICD [13].

The specific criteria used for the diagnosis of pneumonia were that all patients were hospitalized and exhibited the presence of new areas of infiltration on X-ray examination and new clinical symptoms, including at least two of the following: difficulty of breathing, fever > 38°C, sputum production, coughing, and leukocytosis (white blood cell count ≥ 10,000/μL).

Aspiration pneumonia was defined as a form of pneumonia caused by *P*. *aeruginosa* or MDR *P*. *aeruginosa* and triggered by aspiration of foreign bodies, or liquids and foods from the mouth, or vomited gastric contents, or gastric fluid. Aspiration pneumonia was diagnosed based on the medical history, clinical inspection, X-ray examination of the lungs, and finally through the bronchoscopy.

### Tested Antibiotics

The sensitivity and resistance to the following antibiotics were tested against *P*. *aeruginosa*: piperacillin, piperacillin and tazobactam, cefepime, ceftazidime, imipenem, meropenem, ciprofloxacin, levofloxacin, gentamicin, tobramycin, amikacin, fosfomycin, and colistin.

The frequency of use of these antibiotics in clinical practice for the treatment of patients with pneumonia caused by *P*. *aeruginosa* or MDR *P*. *aeruginosa* was recorded. The frequency of testing of these antibiotics on an antibiogram after detecting microbial *Pseudomonas* was noted.

The empiric antibiotic therapy was the initial treatment of pneumonia before the specific bacterium *P*. *aeruginosa* or MDR *P*. *aeruginosa* was identified. It involved administration of commonly used antibiotics based on experience while waiting for the results of the susceptibility testing from the microbiology laboratory.

The inhibition zone diameter breakpoints used for *P*. *aeruginosa* were those recommended in the Clinical and Laboratory Standards Institute 2004 – 2011 guidelines (CLSI, 2012) [14] (Table 1). A change from the CLSI guidelines to Europe-wide standards for susceptibility testing was possible in 2011 because the European Committee on Antimicrobial Susceptibility Testing set Europe-wide standards for susceptibility testing of antibiotics for almost all pathogens on which our tests are based (EUCAST, 2012–2014) [15]. These standards take more clinical and pharmacokinetic aspects of antimicrobial therapy into account. After 2012, therefore, the revised breakpoints based on the EUCAST guidelines were used in the susceptibility testing of each antibiotic (Table 1).

**Table 1:** MIC breakpoints for *Pseudomonas aeruginosa* according to EUCAST and CLSI guidelines. **Abbreviations:** CLSI: Clinical and Laboratory Standards Institute; EUCAST: European Committee on Antimicrobial Susceptibility Testing; MIC: minimum inhibitory concentration.

	EUCAST 2012–2014	CLSI 2004–2011
Antimicrobial	Sensitive ≤ / Resistant > (mg/L)	Sensitive ≤ / Resistant > (μg/mL)
Piperacillin	16 / 16	16/128
Piperacillin + Tazobactam	16 / 16	16/4 / 128/4
Cefepime	8 / 8	8 / 32
Ceftazidime	8 / 8	8 / 32
Imipenem	4 / 8	2 / 8
Meropenem	2 / 8	2 / 8
Ciprofloxacin	0.5 / 1	1 / 4
Levofloxacin	1 / 2	2 / 8
Gentamicin	4 / 4	4 / 16
Tobramycin	4 / 4	4 / 16
Amikacin	8 / 16	16 / 64
Fosfomycin	-/-	32 / 32
Colistin	4 / 4	2 / 8

### Susceptibility testing

*P*. *aeruginosa* colonies from agar plates were suspended in Phoenix ID broth (Becton Dickinson, Heidelberg, Germany) to a 0.5 – 0.6 McFarland standard. Identification was performed either by use of the BD Phoenix^TM^ automated system or by MALDI-TOF MS (Bruker Daltonics, Bremen, Germany). The BD Phoenix^TM^ automated microbiology system (Becton-Dickinson Diagnostic Systems, Sparks, MD, USA), equipped with software suitable for the interpretation of susceptibility testing results using the European Committee on Antimicrobial Susceptibility Testing (EUCAST) breakpoints 2012 – 2014, was used for performing the antimicrobial susceptibility testing EUCAST [15] (Table 1). The panel selected to perform the evaluation was NMIC/ID–76 for Gram-negative bacteria.

The secondary method for susceptibility testing used was the disc diffusion method according to Kirby-Bauer [16]. Disc diffusion was used for screening reasons and in cases where carbapenemase activity had been ruled out, e.g. imipenem, meropenem, or ceftazidime resistance. Synergy testing or a metallo-beta-lactamase E-test was performed when Gram-negative bacterial isolates producing phenotypic metallo-beta-lactamase were suspected.

### Multidrug-resistant *P*. *aeruginosa*

The criterion used in this study for defining MDR in *P*. *aeruginosa* was that *P*. *aeruginosa* was non-susceptible to ≥ 1 agent in ≥ 3 antimicrobial categories in the susceptibility testing of isolates from patients with community-acquired or nosocomial-acquired pneumonia. The antimicrobial categories considered in this investigation were: aminoglycosides; carbapenems; cephalosporins; gyrase inhibitors; penicillin + ß-lactamase inhibitors; epoxide; and polymyxin [17].

### Microbiology

Specimen sites outside the respiratory tract were not pertinent to this study. The isolates belonging to unique patients or repeat patient isolates were excluded. Bronchoalveolar lavage was applied in the context of a bronchoscopy. Tracheal secretions were also collected by fiber-optic bronchoscopy through aspiration into sterile 40 ml specimen traps (Argyle^TM^ Specimen Traps, Covidien Germany Ltd, Neustadt/Donau, Germany). Throat swabs were collected with a commercial cotton swab transport system (MEUS Srl®, Piove di Sacco, Italy) by rotating the swab with slight pressure on the palatal arch of patients with suspected pneumonia. The recovery of sputum was performed by expectoration into a 30 ml sterile sputum collection tube (Salivette®, SARSTEDT, Nümbrecht, Germany).

Microscopy examination of Gram stained material was conducted at 80 – 1,000 fold magnification for at least five visual fields according to the criteria of Bartlett [18].

Columbia Agar with 5% sheep blood (Becton Dickinson, Heidelberg, Germany) was incubated at 37°C for 24 to 48 hours as a general culture medium for the growth and discovery of *Streptococcus pneumoniae*, *Streptococcus pyogenes*, *Staphylococcus aureus*, *Escherichia coli*, and *Shigella flexneri*. BBL^TM^ CHROMagar^TM^ Orientation medium (Becton Dickinson, Heidelberg, Germany) was used for the detection of *Enterobacteriaceae*. The tested *Enterobacteriaceae* were *Escherichia coli*, *Shigella*, *Klebsiella*, *Proteus mirabilis*, *Enterobacter* spp., *Citrobacter* spp., and *Serratia marcescens*. BBL^TM^ CDC Anaerobe 5% Sheep Blood Agar (Becton Dickinson, Heidelberg, Germany) was used for antimicrobial susceptibility testing for the general growth of anaerobes. The different *Pseudomonas* species were identified using MALDI-TOF Biotyper mass spectrometry (Bruker Daltonik Ltd Life Sciences & Chemical Analysis, Bremen, Germany) and the automated microbiology system PHOENIX (Becton Dickinson, Heidelberg, Germany) according to the CLSI guidelines [14].

BD^TM^ Chocolate Agar (Becton Dickinson, Heidelberg, Germany) was used as a variant of blood agar for the isolation and cultivation of *Neisseria* and *Haemophilus* species. BD^TM^ MacConkey Agar (Becton Dickinson, Heidelberg, Germany) was used as a selective medium for the detection of Gram-negative bacteria, especially *Pseudomonas*.

BD^TM^ Pseudosel^TM^ Agar (Cetrimide Agar) was used for the selective isolation and presumptive identification of *P*. *aeruginosa* from clinical specimens [19].

BD^TM^ Sabouraud Agar (Becton Dickinson, Heidelberg, Germany) was used for the cultivation and differentiation of fungi.

### Blood Cultures

At least 20 ml of blood was taken through venipuncture with a blood-collection needle (Safety-Multifly®, SARSTEDT, Nümbrecht, Germany) and injected into two specific media: BACTEC Plus Aerobic/F and Plus Anaerobic/F medium (BD, Becton, Dickinson and Company, Heidelberg, Germany).

### Laboratory

The amounts of C-reactive protein (CRP) in human serum (the normal value is less than 6 mg/L) was measured in lithium heparin SARSTEDT Monovette® 4.7 ml (orange top) using a standard immunoturbidimetric assay on the COBAS® 6000 INTEGRA system c 501 (Roche Diagnostics Ltd, Mannheim, Germany). Leukocyte counts (normal range 4,000 – 10,000/μL) in the blood were generally measured as a routine part of the blood counts after blood collection in EDTA Monovette® (2.7 mL) by flow cytometry using the Sysmex® XE 2100 hematology analyzer (Sysmex Germany Ltd, Norderstedt, Germany).

### Comorbidities

Comorbidities were analyzed in patients with pneumonia caused by *P*. *aeruginosa* or MDR *P*. *aeruginosa*. Comorbidity was considered as the presence of one or more additional disorders existing simultaneously with the primary disease. The additional disorder could be a behavioral or mental disorder.

The length of the hospital stay was also assessed in patients with pneumonia caused by *P*. *aeruginosa* or MDR *P*. *aeruginosa*.

The number of deaths occurring during hospitalization was determined in the study group. The survival analyses were calculated using the Kaplan Meier method.

No experiments in this study were conducted on live vertebrates, including humans. All experiments were performed in accordance with relevant guidelines and regulations of the Witten-Herdecke University, Germany.

### Statistical Analysis

The categorical data were expressed as proportions, while continuous data were expressed as means and standard deviations (SDs). The calculations were performed at 95% confidence intervals (CIs). A chi-square test was carried out for the three probabilities of sensitivity, intermediate sensitivity, and resistance to antibiotics used against *P*. *aeruginosa* or MDR *P*. *aeruginosa* and compared between community-acquired and nosocomial-acquired pneumonia. The chi-square test was also used to compare gender, comorbidities, morbidity, and the various detection methods for *P*. *aeruginosa* or MDR *P*. *aeruginosa* for the two probabilities between community-acquired and nosocomial-acquired pneumonia. One-way analysis of variance (ANOVA) for independent samples was performed to compare age differences, durations of hospital stays, and laboratory tests from the patients with pneumonia caused by *P*. *aeruginosa* or MDR *P*. *aeruginosa*. Two-tailed tests were performed, and a *P* value of < 0.05 was considered statistically significant.

## Results

In the hospital database used in this study, 218 (3.1%) patients were found with pneumonia caused by *P*. *aeruginosa* (ICD J15.1). This is part of a total of 6,932 patients in all age groups with pneumonia caused by different types of bacterial pathogens, who had been treated at the HELIOS Clinic, Witten/Herdecke University, Wuppertal, Germany, during the study period of January 1, 2004 to August 12, 2014.

Fifty patients were excluded from this study. The reasons for the exclusion of these patients were that they had other infectious disease such as urinary infection, urosepsis, gastroenteritis, and meningitis caused by *P*. *aeruginosa* or that access to their patient data at the Department of Neurology was restricted. In addition, patients with pneumonia associated with *P*. *aeruginosa* who were younger than 18 years of age and were treated at the Department of Pediatric and Adolescent Medicine were excluded. Apart from these patients, no other patients were excluded from this study.

A total of 168 (2.4%) of 6,932 patients with a mean age of 68.1 ± 12.8 years [113 (67.3%) males and 55 (32.7%) females] with pneumonia caused by *P*. *aeruginosa* met the inclusion criteria for this trial. The patients were divided into categorical groups depending on the origin of their pneumonia caused by *Pseudomonas*. These groups were 79 (47%) patients with community-acquired, 77 (45.8%) with nosocomial-acquired pneumonia; and 12 (7.1%) with aspiration pneumonia. In general, the number of patients with community-acquired pneumonia due to *P*. *aeruginosa* was higher (91; 54.2%). The average age was higher in patients with nosocomial-acquired pneumonia. Notably, no significant age difference was detected in any of the study groups. Likewise, no gender difference was found between the two study populations. The male sex was more likely to suffer from pneumonia caused by *P*. *aeruginosa* (Table 2).

**Table 2:** Comparison of demographic data, length of hospitalization, and laboratory tests between patients with community-acquired and nosocomial-acquired pneumonia due to *Pseudomonas aeruginosa*. **Abbreviations:** CRP: C-reactive protein; SD: standard deviation.

	Community-acquired pneumonia (n = 91) (%)	Nosocomial-acquired pneumonia (n = 77) (%)	*P value*
Male	59 (64.8)	54 (70.1)	0.572
Female	32 (35.2)	23 (29.9)	0.572
Age mean + SD (years)	66.4 ± 13.8	70.1 ± 11.4	0.062
Duration of hospital stay mean + SD (days)	20.0 ± 23.8	24.2 ± 21.0	0.235
CRP (normal range < 6 mg/L) mean + SD	89.8 ± 93.9	98.3 ± 102.2	0.578
Leukocytes (normal range 4,000 – 10,000/μL) mean + SD	15,350.7 ± 8,421.0	13,450.3 ± 6,835.7	0.118

The average length of the hospital stay was a few days longer for patients with nosocomial-acquired pneumonia caused by *P*. *aeruginosa*, but the difference was not statistically significant (Table 2).

The mean values of CRP were slightly elevated in patients with nosocomial-acquired pneumonia, but the amount of CRP in the serum of patients with community-acquired pneumonia did not differ statistically from that of patients with nosocomial-acquired pneumonia caused by *P*. *aeruginosa* (Table 2). The mean leukocyte counts were slightly increased in patients with community-acquired pneumonia, but they were not statistically different in patients with community-acquired and nosocomial-acquired pneumonia caused by *P*. *aeruginosa* (Table 2).

The susceptibility analysis for each antibiotic varied in this study because some isolates were examined by the automated microbiology system (Becton-Dickinson Diagnostic Systems, Sparks, MD, USA) and others by the disc diffusion method according to Kirby-Bauer. In general, a greater number of tests was performed on the automated microbiology system (Table 3).

**Table 3:** Drug sensitivity and drug resistance in different drug groups of patients with community-acquired compared to nosocomial-acquired pneumonia caused by *Pseudomonas aeruginosa* with MIC_50_ and MIC_90_ breakpoints of each antibiotic. **Abbreviation**: CAP: community-acquired pneumonia; NAP: nosocomial-acquired pneumonia; MIC: minimum inhibitory concentration. **Note:** Significant *P* values are shown in bold.

No. of patients with community-acquired (n = 91) and nosocomial-acquired (n = 77) pneumonia due to *Pseudomonas* aeruginosa (n = 168)										
Drug groups	Active substance	No. using antibiotics (%)	No. of change in antibiotics treatment (n = 33) (%)	No. of tests of antibiotics on antibiogram (%)	Sensitive (%)	Intermediate (%)	Resistant (%)	*P* value Compared CAP + NAP	MIC_50_ (mg/L)	MIC_90_ (mg/L)
Penicillin	Piperacillin	5 (3.0)	5 (15.2)	168 (100)	118 (70.2)	5 (3.0)	45 (26.8)		16	>128
	CAP	3 (60.0)		91 (54.2)	66 (72.5)	3 (3.3)	22 (24.2)	0.698		
	NAP	2 (40.0)		77 (45.8)	52 (67.5)	2 (2.6)	23 (29.9)	0.698		
Penicillin + β-lactamase inhibitors	Piperacillin + Tazobactam	83 (49.4)	3 (9.1)	168 (100)	121 (72.0)	4 (2.4)	43 (25.6)		16	>128
	CAP	49 (59.0)		91 (54.2)	67 (73.6)	3 (3.3)	21 (23.1)	0.533		
	NAP	34 (41.0)		77 (45.8)	54 (70.1)	1 (1.3)	22 (28.6)	0.533		
Cephalosporins	Cefepime	4 (2.4)	2 (6.1)	163 (97.0)	132 (81.0)	4 (2.5)	27 (16.6)			
	CAP	2 (50.0)		90 (55.2)	77 (85.6)	3 (3.3)	10 (11.1)	0.093		
	NAP	2 (50.0)		73 (44.8)	55 (75.3)	1 (1.4)	17 (23.3)	0.093		
	Ceftazidime	10 (6.0)	5 (15.2)	150 (89.3)	114 (76.0)	2 (1.3)	34 (22.7)		4	64
	CAP	3 (30.0)		82 (54.7)	67 (81.7)	2 (2.4)	13 (15.9)	**0.046**		
	NAP	7 (70.0)		68 (45.3)	47 (69.1)	0	21 (30.9)	**0.046**		
Carbapenems	Imipenem	23 (13.7)	2 (6.1)	168 (100)	119 (70.8)	1 (0.6)	48 (28.6)			
	CAP	10 (43.5)		91 (54.2)	64 (70.3)	1 (1.1)	26 (28.6)	0.638		
	NAP	13 (56.5)		77 (45.8)	56 (72.7)	0	21 (27.3)	0.638		
	Meropenem	7 (4.2)	2 (6.1)	163 (97.0)	116 (71.2)	14 (8.6)	33 (20.2)		1	64
	CAP	3 (42.9)		89 (54.6)	65 (73.0)	6 (6.7)	18 (20.2)	0.811		
	NAP	4 (57.1)		74 (45.4)	52 (70.3)	7 (9.5)	15 (20.3)	0.811		
Gyrase inhibitors	Ciprofloxacin	20 (11.9)	11 (33.3)	166 (98.8)	114 (68.7)	2 (1.2)	50 (30.1)		0.25	>4
	CAP	9 (45.0)		91 (54.8)	58 (63.7)	1 (1.1)	32 (35.2)	0.295		
	NAP	11 (55.0)		75 (45.2)	56 (74.7)	1 (1.3)	18 (24.0)	0.295		
	Levofloxacin	6 (3.6)	2 (6.1)	134 (79.8)	53 (39.6)	40 (29.9)	41 (30.6)		0.5	>4
	CAP	4 (66.7)		81 (60.4)	31 (38.3)	22 (27.2)	28 (34.6)	0.440		
	NAP	2 (33.3)		53 (39.6)	22 (41.5)	18 (34.0)	13 (24.5)	0.440		
Amino-glycosides	Gentamicin	6 (3.6)	1 (3.0)	168 (100)	115 (68.5)	16 (9.5)	37 (22.0)			
	CAP	3 (50.0)		91 (54.2)	60 (65.9)	7 (7.7)	24 (26.4)	0.361		
	NAP	3 (50.0)		77 (45.8)	54 (70.1)	9 (11.7)	14 (18.2)	0.361		
	Tobramycin	1 (0.6)	0	139 (82.7)	104 (74.8)	4 (2.9)	31 (22.3)		16	32
	CAP	1 (100)		75 (54.0)	51 (68.0)	3 (4.0)	21 (28.0)	0.194		
	NAP	0		64 (46.0)	52 (81.3)	1 (1.6)	11 (17.2)	0.194		
	Amikacin	0	0	103 (61.3)	83 (80.6)	10 (9.7)	10 (9.7)		4	32
	CAP	0		59 (57.3)	48 (81.4)	5 (8.5)	6 (10.2)	0.878		
	NAP	0		44 (42.7)	35 (79.5)	5 (11.4)	4 (9.1)	0.878		
Epoxide	Fosfomycin	0	0	40 (23.8)	7 (17.5)	0	33 (82.5)		0.5	128
	CAP	0		21 (52.5)	4 (19.0)	0	17 (81.0)	0.966		
	NAP	0		19 (47.5)	3 (15.8)	0	16 (84.2)	0.966		
Polymyxin	Colistin	0	0	9 (5.4)	9 (100)	0	0		0.5	2
	CAP	0		4 (44.4)	4 (100)	0	0	0.574		
	NAP	0		5 (55.6)	5 (100)	0	0	0.574		

Excluding ceftazidime, no significant differences were noted with regard to the susceptibility testing values for any antibiotics for either sensitive, intermediate, or resistant samples determined from the antibiograms of the culture media from the patients with community-acquired compared to nosocomial-acquired pneumonia caused by *P*. *aeruginosa* (Table 3). *P*. *aeruginosa* showed a high resistance rate toward ceftazidime, especially in patients with nosocomial-acquired pneumonia, where the difference was statistically significant (*P* = 0.046) (Table 3).

The most-used antibiotics for the treatment of patients in this study with community-acquired and nosocomial-acquired pneumonia caused by *P*. *aeruginosa* were a combination of piperacillin and tazobactam; imipenem; and ciprofloxacin (Table 3). *P*. *aeruginosa* showed the highest resistance to fosfomycin (Table 3).

Ciprofloxacin was the most frequently changed antibiotic in the treatment of patients with pneumonia based on the antibiotic resistance pattern (Table 3). When necessary, empiric antibiotic therapy was changed; failure of antibiotic treatment was not reported in all patients with pneumonia caused by *P*. *aeruginosa*.

*P*. *aeruginosa* also had a high resistance to ciprofloxacin, levofloxacin, ceftazidime, piperacillin, imipenem, piperacillin and tazobactam, tobramycin, gentamicin, and meropenem (Table 3).

*P*. *aeruginosa* showed no statistical difference in resistance to colistin in either community-acquired or nosocomial-pneumonia (*P* = 0.574; Table 3); it was completely susceptible in both patient populations (100% Table 3). Nevertheless, a low amount of colistin (5.4%, Table 3) was examined in the antibiograms after the detection of *P*. *aeruginosa* in the culture media from tracheal secretions in patients with pneumonia.

**Fig 1:**
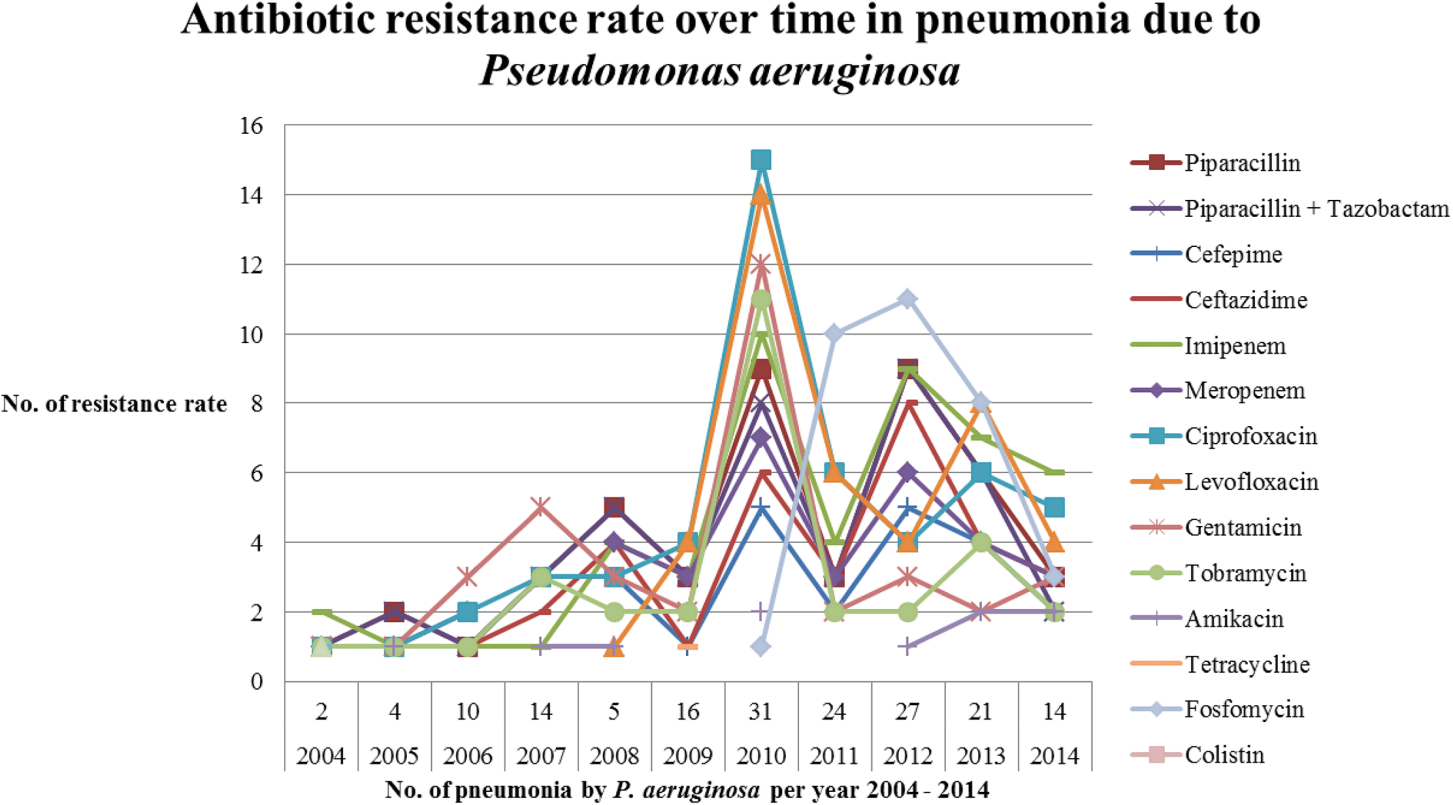
Rate of development of antibiotic resistance over time for pneumonia due to *Pseudomonas aeruginosa* from 2004 to 2014.

Resistance to antibiotics has increased significantly in patients with pneumonia due to *P*. *aeruginosa* over the past years (Fig 1). A peak value of antibiotic resistance was reached for ciprofloxacin (15%), and levofloxacin (14%) in relation to the total sum of 100 cases of all antibiotic resistances in 2010 (Fig 1). In the present study, *P*. *aeruginosa* was most susceptible to the following antibiotics, in order of decreasing effectiveness: cefepime, amikacin, ceftazidime, tobramycin, the combination of piperacillin and tazobactam, meropenem, imipenem, piperacillin, ciprofloxacin, gentamicin, and fosfomycin (Table 3). The ratio of the highest sensitivity of an antibiotic was determined by the amount of each antibiotic that was actually tested.

*P*. *aeruginosa* responded to levofloxacin in susceptibility testing as follows: sensitive (39.6%), intermediate (29.9%), and resistant (30.6%) (Table 3). Therefore, levofloxacin appeared to be poorly effective against *P*. *aeruginosa* for either community-acquired or nosocomial-acquired pneumonia in this study (*P* = 0.440, Table 3).

*P*. *aeruginosa* was most frequently detected in tracheal secretions in both community-acquired and nosocomial-acquired pneumonia, with statistical significance (*P =* 0.034, Table 4).

**Table 4:** The various detection methods used and species of *Pseudomonas aeruginosa* in patients with community-acquired and nosocomial-acquired pneumonia. **Note:** Significant *P* values are shown in bold.

Specimen	Community-acquired pneumonia (n = 91) (%)	Nosocomial-acquired pneumonia (n = 77) (%)	*P value*
Bronchial secretion	26 (28.6)	17 (22.1)	0.435
Tracheal secretion	52 (57.1)	57 (74.0)	**0.034**
Sputum	11 (12.1)	2 (2.6)	**0.045**
Venous blood culture	1 (1.1)	1 (1.3)	0.554
Secretion drainage	1 (1.1)	0	0.920
**Species**			
*Pseudomonas aeruginosa*	91 (100)	77 (100)	

The tracheal and bronchial secretions of patients with pneumonia caused by *Pseudomonas* were sent to the Department of Microbiology at the HELIOS Clinic in Wuppertal, Germany, for further investigation of the microorganisms present in the secretions (Table 4). All *Pseudomonas* species discovered were from isolates of *P*. *aeruginosa* in patients with pneumonia (Table 4).

The most frequently discovered acute comorbidity was cardiac arrhythmia in patients community-acquired and in nosocomial-acquired pneumonia caused by *P*. *aeruginosa* (Table 5). The most common chronic comorbidity was chronic obstructive pulmonary disease in patients with community-acquired and hypertension in nosocomial-acquired pneumonia (Table 5).

**Table 5:** Acute and chronic comorbidities in patients with community-acquired compared to nosocomial-acquired pneumonia caused by *Pseudomonas aeruginosa*. Comorbidities were only considered if they were more than 10% in one of the groups, even if they were less than 10% in the other group. Note: Significant *P* values are shown in bold.

Organ systems	Acute and chronic comorbidities	Community-acquired pneumonia (n = 91) (%)	Nosocomial-acquired pneumonia (n = 77) (%)	*P* value
**Cardiovascular diseases**	***Acute comorbidities***			
	Anemia	19 (20.9)	8 (10.4)	0.102
	Cardiac arrhythmia	23 (25.3)	28 (36.4)	0.165
	Myocardial infarction	7 (7.7)	9 (11.7)	0.538
	Sepsis	8 (8.8)	19 (24.7)	**0.010**
	Shock	4 (4.4)	8 (10.4)	0.229
	***Chronic comorbidities***			
	Coronary artery disease	20 (22.0)	33 (42.9)	**0.006**
	Hypertension	37 (40.7)	37 (48.1)	0.420
	Peripheral arterial occlusive disease	5 ((5.5)	12 (15.6)	0.057
	Valvular heart disease	11 (12.1)	18 (23.4)	0.085
**Pulmonary diseases**	***Acute comorbidities***			
	Acute respiratory failure	13 (14.3)	13 (16.9)	0.807
	Exacerbation by chronic obstructive pulmonary disease	16 (17.6)	2 (2.6)	**0.004**
	Pleural effusion	2 (2.2)	10 (13.0)	**0.016**
	***Chronic comorbidities***			
	Chronic obstructive pulmonary disease	39 (42.9)	13 (16.9)	**0.0005**
	Cor pulmonale	10 (11.0)	5 (6.5)	0.454
**Gastrointestinal diseases**	***Chronic comorbidities***			
	Diabetes	22 (24.2)	17 (22.1)	0.888
	Hyperlipidemia	3 (3.3)	8 (10.4)	0.124
	Obesity	3 (3.3)	9 (11.7)	0.071
	Percutaneous endoscopic gastrostomy	11(12.1)	3 (3.9)	0.102
**Kidney diseases**	***Acute comorbidities***			
	Acute renal failure	7 (7.7)	15 (19.5)	**0.043**
	Acute urinary tract infection	13 (14.3)	6 (7.8)	0.279
	Electrolyte imbalance	10 (11.0)	6 (7.8)	0.663
	***Chronic comorbidities***			
	Chronic renal failure	10 (11.0)	16 (20.8)	0.125
**Neurology diseases**	***Chronic comorbidities***			
	Epilepsy	10 (11.0)	10 (13.0)	0.863
	State after stroke	11 (12.1)	7 (9.1)	0.708

A comparison of the data of patients with community-acquired compared to nosocomial-acquired pneumonia due to MDR *P*. *aeruginosa* revealed no significant differences in the demographic data, length of hospital stay, obtaining of the samples, and the number of leukocytes in the plasma (Table 6). A particularly noticeable effect was the elevated CRP in the plasma of patients with nosocomial-acquired pneumonia due to MDR *P*. *aeruginosa (P =* 0.046, Table 6).

**Table 6:** Comparison of demographic data, length of hospitalization, laboratory tests, specimens, and acute and chronic comorbidities between patients with community-acquired and nosocomial-acquired pneumonia due to multidrug-resistant *Pseudomonas aeruginosa*. **Abbreviations:** CAP: community-acquired pneumonia; NAP: nosocomial-acquired pneumonia; SD: standard deviation.

	Multidrug-resistant *Pseudomonas*		
	CAP (n = 27) (%)	NAP (n = 14) (%)	*P* value
**Age** mean + SD (years)	64.8 ± 18.0	65.1 ± 20.6	1.0
**Gender**			
Male	18 (66.7)	9 (64.3)	0.842
Female	9 (33.3)	5 (35.7)	0.842
**Specimen**			
Bronchial secretion	6 (22.2)	4 (28.6)	1.0
Tracheal secretion	19 (70.4)	9 (64.3)	1.0
Sputum	2 (7.4)	1 (7.1)	0.549
Duration of hospital stay mean + SD (days)	17.5 ± 20.8	25.8 ± 23.6	0.242
CRP (normal range < 6 mg/L) mean + SD	80.3 ± 66.4	131.9 ± 97.6	**0.046**
Leukocytes (normal range 4,000–10,000/μL) mean + SD	13,699.3 ± 5,598.6	11,626.0 ± 6,381.8	0.276
**Acute and chronic comorbidities**			
**Cardiovascular diseases**			
Anemia	8 (29.6)	3 (21.4)	0.842
Bypass surgery	1 (3.7)	2 (14.3)	0.549
Cardiac arrhythmia	8 (29.6)	3 (21.4)	0.842
Cardiac decompensation	3 (11.1)	0	0.507
Coronary artery disease	7 (25.9)	5 (35.7)	0.777
Heart failure	5 (18.5)	1 (7.1)	0.610
Hypertension	10 (37.0)	9 (64.3)	0.183
Valvular heart disease	4 (14.8)	2 (14.3)	0.671
**Pulmonary diseases**			
Acute bronchitis	3 (11.1)	0	0.507
Acute respiratory failure	4 (14.8)	5 (35.7)	0.256
Chronic obstructive pulmonary disease	12 (44.4)	3 (21.4)	0.267
Exacerbation by chronic obstructive pulmonary disease	4 (14.8)	0	0.338
Pleural effusion	1 (3.7)	2 (14.3)	0.549
**Gastrointestinal diseases**			
Diabetes	3 (11.1)	4 (28.6)	0.671
Hyperlipidemia	2 (7.4)	4 (28.6)	0.176
Obesity	1 (3.7)	4 (28.6)	0.071
**Kidney diseases**			
Acute renal failure	2 (7.4)	2 (14.3)	0.888
Acute urinary tract infection	4 (14.8)	2 (14.3)	0.671
Chronic renal failure	3 (11.1)	0	0.507
Electrolyte imbalance	1 (3.7)	2 (14.3)	0.549
**Thyroid disease**			
Hypothyroidism	3 (11.1)	2 (14.3)	0.842
**Neurology diseases**			
Polyneuropathy	3 (11.1)	0	0.584
Epilepsy	1 (3.7)	2 (14.3)	0.549
State after stroke	6 (22.2)	0	0.149
State after encephalopathy	5 (18.5)	0	0.224
Spinal stenosis	0	2 (14.3)	0.212
**Skin disease**			
Pressure ulcer	1 (3.7)	3 (21.4)	0.209

When compared with *P*. *aeruginosa pneumonia*, a greatly increased antibiotic resistance of MDR *P*. *aeruginosa* was found, in decreasing order, for: fosfomycin, ciprofloxacin, levofloxacin, ceftazidime, piperacillin, imipenem, tobramycin, piperacillin-tazobactam, meropenem, cefepime, gentamicin, and amikacin (Table 7). As in the cases of *P*. *aeruginosa* pneumonia, the MDR *P*. *aeruginosa* pneumonia was completely susceptible to colistin, although the number of examined cases of colistin in the antibiograms of patients with pneumonia was relatively low (Table 7). Anemia and chronic obstructive pulmonary disease were the most common comorbidities in patients with community-acquired pneumonia due to MDR *P*. *aeruginosa* (Table 7). Anemia and cardiac arrhythmia were the most common acute comorbidities in patients with community-acquired pneumonia due to MDR *P*. *aeruginosa*, and acute respiratory failure in nosocomial-acquired pneumonia patients caused by MDR *P*. aeruginosa. Chronic obstructive pulmonary disease was the most common chronic comorbidity in patients with community-acquired pneumonia and hypertension in patients with nosocomial-acquired pneumonia due to MDR *P*. *aeruginosa* (Table 6).

**Table 7:** Drug sensitivity and drug resistance in different drug groups of patients with community-acquired compared to nosocomial-acquired pneumonia caused by multidrug-resistant *Pseudomonas aeruginosa*. **Abbreviations:** CAP: community-acquired pneumonia; NAP: nosocomial-acquired pneumonia; MDR Pseudomonas: multidrug resistant *Pseudomonas*. **Note:** Significant *P* values are shown in bold.

		MDR *Pseudomonas* CAP (n = 27), NAP (n = 14)								
Drug groups	Active substance	No. of tests of antibiotics on antibiogram		Sensitive		Intermediate		Resistant		ComparisonCAP + NAP
		CAP	NAP	CAP (%)	NAP (%)	CAP(%)	NAP (%)	CAP (%)	NAP (%)	*P* value
Penicillin	Piperacillin	27	14	15 (55.6)	6 (35.3)	0	0	12 (44.4)	8 (57.1)	0.741
Penicillin + β-lactamase inhibitors	Piperacillin + Tazobactam	27	14	15 (55.6)	7 (50.0)	0	0	12 (44.4)	7 (50.0)	0.947
Cephalosporins	Cefepime	27	14	20 (74.1)	7 (50.0)	0	0	7 (25.9)	7 (50.0)	0.304
	Ceftazidime	27	13	16 (59.3)	5 (38.5)	2 (7.4)	0	9 (33.3)	8 (61.5)	0.189
Carbapenems	Imipenem	27	14	11 (40.7)	7 (50.0)	1 (3.7)	0	15 (55.6)	7 (50.0)	0.687
	Meropenem	26	14	12 (46.2)	6 (42.9)	3 (11.5)	1 (7.1)	11 (42.3)	7 (50.0)	0.852
Gyrase inhibitors	Ciprofloxacin	27	13	8 (29.6)	4 (30.8)	0	1 (7.7)	19 (70.4)	8 (61.5)	0.336
	Levofloxacin	27	14	6 (22.2)	3 (21.4)	3 (11.1)	2 (14.3)	18 (66.7)	9 (64.3)	0.956
Aminoglycosides	Gentamicin	27	14	15 (55.6)	10 (71.4)	0	1 (7.1)	12 (44.4)	3 (21.4)	0.162
	Tobramycin	25	12	11 (44.0)	9 (75.0)	1 (4.0)	0	13 (52.0)	3 (27.3)	0.193
	Amikacin	22	11	14 (63.6)	10 (90.9)	2 (9.1)	0	6 (27.3)	1 (9.1)	0.236
Epoxide	Fosfomycin	7	7	2 (28.6)	1 (14.3)	5 (71.4)	0	0	6 (85.7)	**0.0035**
Polymyxin	Colistin	4	2	4 (100)	2 (100)	0	0	0	0	1.0

A total of 33 (19.6%) deaths occurred: 14 (15.4%) patients died from community-acquired pneumonia caused by *P*. *aeruginosa* and 2 (7.4%) due to MDR *P*. *aeruginosa*. Most deaths were in the group with nosocomial-acquired pneumonia caused by *P*. *aeruginosa*. Nineteen (24.7%) patients died from nosocomial-acquired pneumonia caused by *P*. *aeruginosa* and 4 (28.6%) due to MDR *P*. *aeruginosa*. Overall, 9.3% more deaths occurred due to nosocomial-acquired pneumonia caused by *P*. *aeruginosa* (*P* = 0.188) and 21.2% more deaths occurred due to nosocomial-acquired pneumonia due to MDR *P*. *aeruginosa* (*P* = 0.176), but these differences were not statistically significant. Thus, the survival rate in this study was significantly lower in patients with nosocomial-acquired pneumonia caused by *P*. *aeruginosa*, at 75.3% (95% CI 64.2% – 86.4%), and MDR *P*. *aeruginosa*, at 71.4% (95% CI 43.4%– 98.4%), compared to those with community-acquired pneumonia caused by *P*. *aeruginosa*, at 84.6% (95% CI 76.6% – 92.7%), and MDR *P*. *aeruginosa*, at 92.6% (95% CI 82.3%– 102.9%).

## Discussion

During the ten years of this qualitative control observational study, not all antibiotics were tested with the same frequency, either manually or with the Phoenix automated system, after microbiological isolation of *P*. *aeruginosa* isolates from respiratory specimens of patients with pneumonia at the Department of Microbiology.

Fosfomycin is an effective treatment of uncomplicated urinary tract and gastrointestinal infections against both Gram-positive and Gram-negative bacteria. Therefore, fosfomycin has recently been recommended as an intensive care treatment for *P*. *aeruginosa* pneumonia in patients with chronic respiratory diseases [20,21]. However, the present study revealed a high resistance of *P*. *aeruginosa* to fosfomycin after examining the antibiograms of patients with pneumonia.

Surprisingly, the antimicrobial susceptibility testing of the *P*. *aeruginosa* isolates from the tracheal secretions performed in this study also revealed that colistin was the only antibiotic that showed no resistance over the years, although the number of *P*. *aeruginosa* isolates tested for colistin was rather low. Nevertheless, colistin has been increasingly used for the treatment of multi-resistant Gram-negative infections. Little recent data are available regarding the susceptibility of Gram-negative bacteria to colistin, in part because susceptibility testing for colistin remains problematic and also because the use of colistin is not widespread. Colistin resistance is uncommon in *Enterobacteriaceae*. The results of a previous study, as well as the present one, indicate that susceptibility testing should be performed each time the clinical use of the colistin is considered [22].

Another past study that evaluated the in vitro activity of colistin in isolates of Gram-negative bacteria using Clinical and Laboratory Standards Institute broth micro-dilution methods reported that all MDR *P*. *aeruginosa* isolates were susceptible to colistin. These data support a role for colistin in the treatment of infections caused by MDR *P*. *aeruginosa* [23]. However, the emergence of resistance during monotherapy is a concern, so colistin should be combined with other treatments for *P*. *aeruginosa* pneumonia. This practice should be advocated especially in multidrug-resistant cases. Combination regimens could include imipenem, meropenem, aztreonam, piperacillin, ceftazidime, or ciprofloxacin, but none of these regimens have shown any improved outcome in clinical studies [24]. Prolonged colistin exposure (e.g., 2 weeks or longer) may be a prerequisite for resistance development in particular *P*. *aeruginosa* strains. Ciprofloxacin might not be a wise empiric therapy choice, especially in critical infections, due to its rising resistance (24% or higher), as was observed in the present study over the years.

In contrast to the data in the literature, *P*. *aeruginosa* caused more community-acquired pneumonia than nosocomial-acquired pneumonia in the current study [25]. Due to the high resistance of *P*. *aeruginosa* to levofloxacin, a combination therapy of levofloxacin with gentamicin is recommended for the treatment of pneumonia caused by *P*. *aeruginosa* [26]. *P*. *aeruginosa* showed no effect of levofloxacin in susceptibility testing in this study. Therefore, levofloxacin is not recommended as a monotherapy for the treatment of pneumonia due to *Pseudomonas*.

Until now, ceftazidime was held as the most effective antibiotic among the cephalosporin group for the treatment of pneumonia due to *P*. *aeruginosa* [27]. However, ceftazidime showed low activity against *Pseudomonas* in the present investigation.

A past study found a similar resistance of *P*. *aeruginosa* to piperacillin to that found in the present study [28]. In general, piperacillin has the broadest spectrum of activity of all penicillins against *Pseudomonas*. However, the combination therapy of piperacillin with a β-lactamase inhibitor tazobactam to improve the effectiveness revealed an increased resistance of *P*. *aeruginosa* to even this combination of antibiotics in the present study. This antibiotic combination is often used for the treatment of severe pneumonia due to *P*. *aeruginosa* in critically ill patients [29]. However, treatment with piperacillin-tazobactam is now controversial due to the reduced susceptibility of *Pseudomonas* [30]. A reduced effectiveness of piperacillin-tazobactam, which was the most commonly used antibiotic and the most frequently tested on patient antibiograms, was also observed in patients with pneumonia due to *P*. *aeruginosa* in the present study.

Due to their very broad spectrum of activity, carbapenems are also effective against *P*. *aeruginosa*. Therefore, imipenem and meropenem are beneficial antibiotics for the treatment of pneumonia due to *P*. *aeruginosa*. However, metallo-β-lactamases hydrolyze this antibiotic class quite efficiently [31]. Therefore, immediate detection of metallo-β-lactamase-producing *P*. *aeruginosa* is essential for accurate treatment of pneumonia caused by this bacterium. Development of carbapenem resistance was also evident for pneumonia due *P*. *aeruginosa* in this study.

Tobramycin has a narrow spectrum of activity, but it is often used to eliminate *P*. *aeruginosa* in patients with cystic fibrosis [32]. This aminoglycoside antibiotic is commonly used to treat different Gram-negative bacteria [33]. It is principally effective against *P*. *aeruginosa*. Despite the antipseudomonal effectiveness of tobramycin, an increased resistance of *P*. *aeruginosa* to tobramycin was evident in the susceptibility testing performed in the present study.

An increased resistance of *P*. *aeruginosa* to gentamicin was also seen in this study. This antibiotic is often used clinically in combination with beta-lactams or cephalosporins for the treatment of serious pneumonia caused by *P*. *aeruginosa* [34].

Cefepime is one of the few antibiotics described to have constant antipseudomonal activity over the years, although publications on cefepime resistance are growing in number in recent years [35,36]. An increasing resistance of *P*. *aeruginosa* to this cephalosporin was also detected in the present investigation.

Amikacin has activity against *P*. *aeruginosa*, but the combination of cefepime and amikacin was reported as more effective than cefepime monotherapy in the treatment of nosocomial-acquired pneumonia due to *P*. *aeruginosa* [37]. Although this antibiotic showed the lowest resistance among the examined antibiotics, a resistance of *P*. *aeruginosa* to this aminoglycoside was noted in the present study.

Unfortunately, pneumonia due to *Pseudomonas* is becoming more challenging to treat because of growing antibiotic resistance. An increase in the incidence of MDR *P*. *aeruginosa* pneumonia was also observed over the years in this study. The precise incidence of MDR *P*. *aeruginosa* is not known [38], but several reasons probably explain its appearance. First, a discrepancy may have arisen due to different definitions of MDR *P*. *aeruginosa* used in the past studies [39]. The definition of the resistance of MDR *P*. *aeruginosa* ranges in the literature from one antibiotic to all tested antibiotics [39]. Furthermore, no international monitoring systems have been established for MDR *P*. *aeruginosa* [38]. The monitoring of the exact prevalence of MDR *P*. *aeruginosa* is further complicated by the annual variation in prevalence in different geographical areas [38].

The average age of patients with MDS *P*. *aeruginosa* pneumonia in the present study was slightly higher compared to *P*. *aeruginosa*. The elderly population is particularly vulnerable to *Pseudomonas* pneumonia as a result of increased comorbidities such as diabetes mellitus and chronic lung diseases [40]. These two diseases were increasingly seen in elderly people with MDR *P*. *aeruginosa* pneumonia in the present study. A meta-analysis of data from past studies demonstrated a statistically non-significant increased risk of mortality in patients with pneumonia due to resistant *P*. *aeruginosa* [41]. An increased mortality of patients with pneumonia due to MDR *P*. *aeruginosa* was also noticed in this study. Similar to the results reported in an earlier study [42], MDR *P*. *aeruginosa* was increasingly discovered in male patients with pneumonia in the present study, although no significant statistical difference was noted between the sexes in either study.

The levels of CRP were elevated in the plasma of patients with pneumonia caused by MDR *P*. *aeruginosa* in the present study. The CRP level used as a follow-up parameter for pneumonia in addition to the clinical parameters. The monitoring of pneumonia with CRP has been validated as a follow-up during treatment of pneumonia with antibiotics [43].

### Study Limitations

This study describes the situation of *P*. *aeruginosa* resistance in a single hospital, so the results cannot be generalized to other geographic locations. The data in this study are mainly relevant for this single institution. Evaluation of the study findings revealed that not all antibiotics were tested with the same frequency in the antibiograms of patients with pneumonia caused by *P*. *aeruginosa*. This study was unable to clarify whether all of these antibiotic substances had been tested on each antibiogram or had been detailed in the diagnosis of susceptibility testing.

## Conclusions

Single-drug or multidrug resistance in *P*. *aeruginosa* isolates is an everyday reality for laboratories and hospitals and evidence is growing regarding the ability of certain resistant clones to spread by cross-transmission. This study represents an attempt to cover resistant infections from a small evidence base and with the threat of limited new drugs in the pipeline. All patients with pneumonia caused by *P*. *aeruginosa* showed resistance to various antimicrobial agents. Few *P*. *aeruginosa* isolates were fully susceptible to colistin. This finding points to the importance of studying the median duration of antimicrobial treatment in correlation to low mortality rates in pneumonia patients and of intensifying these trials in terms of differences in empiric therapy and definitive therapy as single drugs or in combination. Further studies should focus on better administration of the existing antibiotic armamentarium, along with antimicrobial stewardship programs, and should fully support the quest for new antibiotics.

## References

[1] de Bentzmann S, Plésiat P. The *Pseudomonas aeruginosa* opportunistic pathogen and human infections. Environ. Microbiol. 2011; 13: 1655–1665. 10.1111/j.1462-2920.2011.02469.x 21450006

[2] Hardalo C, Edberg SC. *Pseudomonas aeruginosa*: assessment of risk from drinking water. Crit. Rev. Microbiol. 1997; 23: 47–75. 9097014 10.3109/10408419709115130

[3] Mena KD, Gerba CP. Risk assessment of Pseudomonas aeruginosa in water. Rev Environ Contam. Toxicol. 2009 201, 71–115. 10.1007/978-1-4419-0032-6_3 19484589

[4] Zarb P, Coignard B, Griskeviciene J, Muller A, Vankerckhoven V, Weist K, et al The European Centre for Disease Prevention and Control (ECDC) pilot point prevalence survey of healthcare-associated infections and antimicrobial use. Euro Surveill. 2012; 17: 20316 23171822 10.2807/ese.17.46.20316-en

[5] Kollef MH, Chastre J, Fagon JY, François B, Niederman MS, Rello J, et al Global prospective epidemiologic and surveillance study of ventilator-associated pneumonia due to Pseudomonas aeruginosa. Crit. Care Med. 2014; 42:2178–87. 10.1097/CCM.0000000000000510 25054674

[6] Bubonja-Sonje M, Matovina M, Skrobonja I, Bedenic B, Abram M. Mechanisms of Carbapenem Resistance in Multidrug-Resistant Clinical Isolates of Pseudomonas aeruginosa from a Croatian Hospital. Microb Drug Resist. 2015; [Epub ahead of print]. 10.1089/mdr.2014.0172 25565041

[7] Tran CS, Rangel SM, Almblad H, Kierbel A, Givskov M, Tolker-Nielsen T, et al The Pseudomonas aeruginosa type III translocon is required for biofilm formation at the epithelial barrier. PLoS Pathog. 2014; 10: e1004479 10.1371/journal.ppat.1004479 25375398 PMC4223071

[8] Hancock RE, Speert DP. Antibiotic resistance in Pseudomonas aeruginosa: mechanisms and impact on treatment. Drug. Resist. Updat. 2000; 3: 247–255. 11498392 10.1054/drup.2000.0152

[9] Akter S, Shamsuzzaman SM, Jahan F. Community acquired bacterial pneumonia: aetiology, laboratory detection and antibiotic susceptibility pattern. Malays. J. Pathol. 2014; 36: 97–103. 25194532

[10] Aloush V, Navon-Venezia S, Seigman-Igra Y, Cabili S, Carmeli Y. Multidrug-resistant *Pseudomonas aeruginosa*: risk factors and clinical impact. Antimicrob. Agents Chemother. 2006; 50: 43–48. 16377665 10.1128/AAC.50.1.43-48.2006 PMC1346794

[11] Niederman MS, Mandell LA, Anzueto A, Bass JB, Broughton WA, Campbell GD, et al Guidelines for the management of adults with community-acquired pneumonia. Diagnosis, assessment of severity, antimicrobial therapy, and prevention. Am. J. Respir. Crit. Care Med. 2001; 163: 1730–1754. 11401897 10.1164/ajrccm.163.7.at1010

[12] Watkins RR, Lemonovich TL. Diagnosis and management of community-acquired pneumonia in adults. Am. Fam. Physician. 2011; 83: 1299–1306. 21661712

[13] World Health Organization (WHO). International Classification of Diseases (ICD). Available: http://www.who.int/classification/icd/en/. Accessed 23 January 2015.

[14] Clinical and Laboratory Standards Institute. Performance standards for antimicrobial susceptibility testing. CLSI M100-S22. Available: http://clsi.org/blog/2012/01/13/clsi-publishes-2012-antimicrobial-susceptibility-testing-standards/. Accessed 23 January 2015.

[15] European Committee on Antimicrobial Susceptibility Testing (EUCAST) breakpoints 2011–2014. Available: http://www.eucast.org. Accessed 23 January 2015.

[16] Bauer AW, Kirby WM, Sherris JC, Turck M. Antibiotic susceptibility testing by a standardized single disk method. Am. J. Clin. Pathol. 1966; 45: 493–496. 5325707

[17] Magiorakos AP, Srinivasan A, Carey RB, Carmeli Y, Falagas ME, Giske CG, et al Multidrug-resistant, extensively drug-resistant and pandrug-resistant bacteria: an international expert proposal for interim standard definitions for acquired resistance. Clin. Microbiol. Infect. 2012; 18: 268–281. 10.1111/j.1469-0691.2011.03570.x 21793988

[18] Barlett JG. Diagnosis of bacterial infections of the lung. Clin Chest Med. 1987; 8, 119–134. 3552385

[19] Pseudosel™ Agar (Cetrimide Agar). Available: http://www.bd.com/ds/productCenter/297882.asp. Accessed 23 January 2015.

[20] Falagas ME, Giannopoulou KP, Kokolakis GN, Rafailidis PI. Fosfomycin: use beyond urinary tract and gastrointestinal infections. Clin. Infect. Dis. 2008; 46: 1069–1077. 10.1086/527442 18444827

[21] Shorr AF. Review of studies of the impact on Gram-negative bacterial resistance on outcomes in the intensive care unit. Crit. Care Med. 2009; 37: 1463–1469. 10.1097/CCM.0b013e31819ced02 19242341

[22] Tan TY, Ng SY. The in-vitro activity of colistin in gram-negative bacteria. Singapore Med. J. 2006; 47: 621–624. 16810437

[23] Walkty A, DeCorby M, Nichol K, Karlowsky JA, Hoban DJ, Zhanel GG. In vitro activity of colistin (polymyxin E) against 3,480 isolates of gram-negative bacilli obtained from patients in Canadian hospitals in the CANWARD study, 2007–2008. Antimicrob. Agents Chemother. 2009; 53: 4924–4926. 10.1128/AAC.00786-09 19704135 PMC2772319

[24] Petrosillo N, Ioannidou E, Falagas ME. Colistin monotherapy vs. combination therapy: evidence from microbiological, animal and clinical studies. Clin. Microbiol. Infect. 2008; 14: 816–827. 10.1111/j.1469-0691.2008.02061.x 18844682

[25] Fujitani S, Sun HY, Yu VL, Weingarten JA. Pneumonia due to Pseudomonas aeruginosa: part I: epidemiology, clinical diagnosis, and source. Chest. 2011; 139: 909–919. 10.1378/chest.10-0166 21467058

[26] Noreddin AM, Elkhatib WF. Levofloxacin in the treatment of community-acquired pneumonia. Expert Rev Anti Infect Ther. 2010;8:505–514. 10.1586/eri.10.35 20455679

[27] Castanheira M, Mills JC, Farrell DJ, Jones RN. Mutation-driven β-lactam resistance mechanisms among contemporary ceftazidime-nonsusceptible *Pseudomonas aeruginosa* isolates from U.S. hospitals. Antimicrob Agents Chemother. 2014; 58: 6844–6850. 10.1128/AAC.03681-14 25182652 PMC4249397

[28] Riou M, Carbonnelle S, Avrain L, Mesaros N, Pirnay JP, Bilocq F, et al In vivo development of antimicrobial resistance in *Pseudomonas aeruginosa* strains isolated from the lower respiratory tract of Intensive Care Unit patients with nosocomial pneumonia and receiving antipseudomonal therapy. Int J Antimicrob Agents. 2010; 36: 513–522. 10.1016/j.ijantimicag.2010.08.005 20926262

[29] Lodise TP Jr, Lomaestro B, Drusano GL. Piperacillin-tazobactam for Pseudomonas aeruginosa infection: clinical implications of an extended-infusion dosing strategy. Clin Infect Dis. 2007; 44: 357–363 17205441 10.1086/510590

[30] Tam VH, Gamez EA, Weston JS, Gerard LN, Larocco MT, Caeiro JP, et al Outcomes of bacteremia due to *Pseudomonas aeruginosa* with reduced susceptibility to piperacillin-tazobactam: implications on the appropriateness of the resistance breakpoint. Clin Infect Dis. 2008; 46: 862–867. 10.1086/528712 18279040

[31] Farajzadeh Sheikh A, Rostami S, Jolodar A, Tabatabaiefar MA, Khorvash F, Saki A, et al Detection of metallo-beta lactamases among carbapenem-resistant *Pseudomonas aeruginosa* . Jundishapur J Microbiol. 2014; 7: e12289 10.5812/jjm.12289 25774271 PMC4332233

[32] Somayaji R, Parkins MD. Tobramycin inhalation powder: an efficient and efficacious therapy for the treatment of Pseudomonas aeruginosa infection in cystic fibrosis. Ther Deliv. 2015; 6: 121–137. 10.4155/tde.14.94 25690082

[33] Bulitta JB, Ly NS, Landersdorfer CB, Wanigaratne NA, Velkov T, Yadav R, et al Two mechanisms of killing of *Pseudomonas aeruginosa* by tobramycin assessed at multiple inocula via mechanism-based modeling. Antimicrob Agents Chemother. 2015; 59: 2315–2327. 10.1128/AAC.04099-14 25645838 PMC4356757

[34] Tam VH, Kabbara S, Vo G, Schilling AN, Coyle EA. Comparative pharmacodynamics of gentamicin against Staphylococcus aureus and Pseudomonas aeruginosa. Antimicrob Agents Chemother. 2006; 50: 2626–2631. 16870751 10.1128/AAC.01165-05 PMC1538660

[35] Sader HS, Fritsche TR, Jones RN. Potency and spectrum trends for cefepime tested against 65,746 clinical bacterial isolates collected in North American medical centers: results from the SENTRY Antimicrobial Surveillance Program (1998–2003) Diagn Microbiol Infect Dis. 2005; 52: 265–273. 16105569 10.1016/j.diagmicrobio.2005.02.003

[36] Kotwal A, Biswas D, Kakati B, Thakuria B, Bhardwaj N. Efficacy of anti-pseudomonal antibiotics: need to reconsider the empirical use of cefepime. Indian J Med Res. 2014; 140: 560–562. 25488453 PMC4277145

[37] Chaudhary M, Shrivastava SM, Varughese L, Sehgal R. Efficacy and safety evaluation of fixed dose combination of cefepime and amikacin in comparison with cefepime alone in treatment of nosocomial pneumonia patients. Curr Clin Pharmacol. 2008; 3:118–122. 18700304 10.2174/157488408784293660

[38] Hirsch EB, Tam VH. Impact of multidrug-resistant *Pseudomonas aeruginosa* infection on patient outcomes. Expert Rev Pharmacoecon Outcomes Res. 2010; 10: 441–451. 10.1586/erp.10.49 20715920 PMC3071543

[39] Falagas ME, Koletsi PK, Bliziotis IA. The diversity of definitions of multidrug-resistant (MDR) and pandrug-resistant (PDR) *Acinetobacter baumannii* and *Pseudomonas aeruginosa* . J Med Microbiol. 2006; 55: 1619–1629. 17108263 10.1099/jmm.0.46747-0

[40] Pappas G, Saplaoura K, Falagas ME. Current treatment of pseudomonal infections in the elderly. Drugs Aging. 2009; 26: 363–79. 10.2165/00002512-200926050-00001 19552489

[41] Nathwani D, Raman G, Sulham K, Gavaghan M, Menon V. Clinical and economic consequences of hospital-acquired resistant and multidrug-resistant *Pseudomonas aeruginosa* infections: a systematic review and meta-analysis. Antimicrob Resist Infect Control. 2014; 3: 32 10.1186/2047-2994-3-32 25371812 PMC4219028

[42] Aloush V, Navon-Venezia S, Seigman-Igra Y, Cabili S, Carmeli Y. Multidrug-resistant *Pseudomonas aeruginosa*: risk factors and clinical impact. Antimicrob Agents Chemother. 2006; 50: 43–8. 16377665 10.1128/AAC.50.1.43-48.2006 PMC1346794

[43] Bruns AH, Oosterheert JJ, Hak E, Hoepelman AI. Usefulness of consecutive C-reactive protein measurements in follow-up of severe community-acquired pneumonia. Eur Respir J. 2008; 32: 726–32. 10.1183/09031936.00003608 18508833

